# Cooling Uncouples Differentially ROS Production from Respiration and Ca^2+^ Homeostasis Dynamic in Brain and Heart Mitochondria

**DOI:** 10.3390/cells11060989

**Published:** 2022-03-14

**Authors:** Neven Stevic, Jennifer Maalouf, Laurent Argaud, Noëlle Gallo-Bona, Mégane Lo Grasso, Yves Gouriou, Ludovic Gomez, Claire Crola Da Silva, René Ferrera, Michel Ovize, Martin Cour, Gabriel Bidaux

**Affiliations:** 1 Univ-Lyon, CarMeN Laboratory, Inserm U1060, Université Claude Bernard Lyon 1, INSA Lyon, F-69550 Bron, France; neven.stevic@chu-lyon.fr (N.S.); jennifer_maalouf21@hotmail.com (J.M.); laurent.argaud@chu-lyon.fr (L.A.); noelle.gallo-bona@univ-lyon1.fr (N.G.-B.); megane.lo-grasso@inserm.fr (M.L.G.); yves.gouriou@univ-lyon1.fr (Y.G.); ludovic.gomez@inserm.fr (L.G.); claire.crola-da-silva@univ-lyon1.fr (C.C.D.S.); rene.ferrera@univ-lyon1.fr (R.F.); michel.ovize@gmail.com (M.O.); martin.cour@chu-lyon.fr (M.C.); 2 Hospices Civils de Lyon, Groupement Hospitalier EST, Département de Cardiologie, IHU-OPERA Bâtiment B13, F-69500 Bron, France; 3 Hospices Civils de Lyon, Hôpital Edouard Herriot, Service de Médecine Intensive-Réanimation, F-69437 Lyon, France

**Keywords:** cold, brain mitochondria, heart mitochondria, mitochondrial functions, mitochondrial respiration, Ca^2+^ retention capacity, MCU, reactive oxygen species, reverse electron transport, ischemia/reperfusion

## Abstract

Hypothermia provides an effective neuro and cardio-protection in clinical settings implying ischemia/reperfusion injury (I/R). At the onset of reperfusion, succinate-induced reactive oxygen species (ROS) production, impaired oxidative phosphorylation (OXPHOS), and decreased Ca^2+^ retention capacity (CRC) concur to mitochondrial damages. We explored the effects of temperature from 6 to 37 °C on OXPHOS, ROS production, and CRC, using isolated mitochondria from mouse brain and heart. Oxygen consumption and ROS production was gradually inhibited when cooling from 37 to 6 °C in brain mitochondria (BM) and heart mitochondria (HM). The decrease in ROS production was gradual in BM but steeper between 31 and 20 °C in HM. In respiring mitochondria, the gradual activation of complex II, in addition of complex I, dramatically enhanced ROS production at all temperatures without modifying respiration, likely because of ubiquinone over-reduction. Finally, CRC values were linearly increased by cooling in both BM and HM. In BM, the Ca^2+^ uptake rate by the mitochondrial calcium uniporter (MCU) decreased by 2.7-fold between 25 and 37 °C, but decreased by 5.7-fold between 25 and 37 °C in HM. In conclusion, mild cold (25–37 °C) exerts differential inhibitory effects by preventing ROS production, by reverse electron transfer (RET) in BM, and by reducing MCU-mediated Ca^2+^ uptake rate in BM and HM.

## 1. Introduction

Cold has been used for decades for neuroprotection and cardioprotection against ischemia-reperfusion injury [1,2,3]. In the clinical field, targeted temperatures range from 4–8 °C (deep) in graft preservation and cardioplegia to 32–36 °C (mild) in post-cardiac arrest care [2,4]. In some cardiac surgeries, core temperature is also decreased as low as 20 °C, mostly for brain protection. Despite intense research, the underlying protective mechanisms of therapeutic hypothermia remain poorly understood [5].

Mitochondrion plays a central role in ischemia-reperfusion injury. Indeed, at the onset of reperfusion, cells can undergo a harmful mitochondrial Ca^2+^ overload, facilitating the opening of the mitochondrial permeability transition pore (mPTP). This is thought to be concomitant to a burst of mitochondrial ROS production at the restart of the respiratory chain [6]. Chouchani et al. have reported that succinate accumulation during ischemia could play a central role in this reperfusion ROS burst. They proposed this latter was induced via the reverse electron transport (RET) towards complex I [7]. However, an interesting alternative hypothesis involves the over-reduction in the ubiquinone (CoQ) pool through electrons coming from both complex I and II [8]. Over-reduced CoQ would create a bottleneck for electron, thus increasing electron leak through ROS production by Complex I. All these mechanisms, in addition to the energetic fall during ischemia, concur to the mPTP opening that causes a massive release of Ca^2+^ contained inside mitochondria and can finally lead to cell death [9,10]. Studies exploring those functions according to temperature are scarce.

Cold influence on mitochondria functions may go far beyond a simple slowdown in chemical reactions, as formalized in the Arrhenius law [11], and studies have reported non-linear effects of cold within the range of 5–37 °C [12,13]. Accumulating data suggest that the hypothermia protective effect could be linked to modifications of the respiratory chain and mitochondrial ROS production [5,13]. However, to our knowledge, only a few studies have taken into account the influence of mitochondrial chain substrates, especially succinate, and the modifications of the ratio between succinate and malate/glutamate, which could also alter mitochondrial function after ischemia-reperfusion and response to cold [7]. No studies have focused on the effect of cold on mitochondrial activity and the subsequent mPTP opening, which is a major event at reperfusion [14,15]. Finally, since it is usually thought that cold-response is a ubiquitous phenomenon, there have been no studies comparing the functions of isolated mitochondria from the brain and heart.

The present study investigated the relationship between a range of temperatures (6, 10, 15, 20, 25, 31, and 37 °C) and several mitochondrial functions—namely, respiration, ROS production and CRC—in brain and heart mitochondria isolated from wild-type mouse. We confirmed that, in both organs, the relationship between temperature and activities of the respiratory chain (O_2_ consumption and ROS production) is non-linear with a temperature coefficient (Q_10_) in the range of Arrhenius values. More interestingly, we found out that temperature affects, non-linearly, both mitochondrial Ca^2+^ influx via the mitochondrial Ca^2+^ uniporter (MCU) and steady-state Ca^2+^ concentration outside of mitochondria though a mitochondrial Na^+^/Ca^2+^ exchanger (NCLX)-dependent mechanism in both organs. Finally, we emphasized strong difference in the behavior of HM as compared to BM.

## 2. Material and Methods

### 2.1. Animals

The study was conducted according to the guidelines of the Declaration of Helsinki, and approved by the local institutional animal research committee (N° BH2012-65 for the surgical procedure and N° BH2012-64 for brain and heart collect). C57BL/6J male mice, aged between 12 and 16 weeks, were obtained from Charles River Laboratory. They were continuously exposed to light, with free access to food and water. They received human care according to the NIH Guide on the Use of Laboratory Animals (NIH Publication No. 85-23, revised 2011).

### 2.2. Mitochondria Extraction

After euthanasia of animal by cervical dislocation, brain and heart mitochondrial fractions were isolated by differential centrifugation. All operations were carried out in the cold or on ice. Brain or heart tissue was placed in a cold isolation buffer (75 mM sucrose, 225 mM mannitol, 0.1 mM EGTA, 20 mM HEPES/Tris, pH 7.40), finely minced and then homogenized in the same buffer (18 mL/g) with 5 mg/mL BSA, using a Potter Elvehjem. For the heart tissue only, the sample was incubated for 1 min with proteases (3 U/g of heart tissue). The homogenate was first centrifuged at 500× *g* for 5 min, then the supernatant was centrifuged at 1000× *g* for 5 min (this step was performed only for brain mitochondria), and this last centrifugation supernatant was centrifuged at 9000× *g* for 10 min. The mitochondrial pellet was suspended and homogenized in the same buffer without Ca^2+^ chelating agent and with 10 mM HEPES/Tris. Mitochondria were kept on ice until use and were assayed within 4 h of isolation.

### 2.3. Protein and Mitochondria Quantification

Protein concentration was used to estimate mitochondria concentration in each sample. Samples containing 500 µg of proteins for brain and 250 µg for heart were used for experiments. Protein content was then assayed according to the Lowry method [16] using BSA as a standard; absorbance was read at 620 nm on a Spark multimode microplate reader (Tecan Trading AG, Männedorf, Switzerland).

Quantification of mitochondrial count was achieved by flow cytometry (BD LFortessa X-20, BD Biosciences, Franklin Lakes, NJ, USA) and the protein concentration was adjusted for brain and heart to 500 µg and 250 µg, respectively. In this control experiment, mitochondria were identified by nonyl acridine orange (NAO) staining. Brain and heart mitochondria (50 µg of total proteins) were counted under flow setting at 12 µL/min. Brain mitochondria exhibited different morphological characteristics from heart mitochondria, as represented on the histograms (Supplemental Data Figure S1A) by the size (forward scatter) and the granularity (side scatter) of mitochondria populations. This 50 µg of brain and heart samples contained 21 × 10^6^ and 32 × 10^6^ mitochondria, respectively (Supplemental Data Figure S1B). Consequently, a 500 µg brain sample for biochemical analysis contained 1.31 times more mitochondria than a 250 µg heart sample.

### 2.4. Mitochondrial Oxygen Consumption

Oxygen consumption was quantified to report the mitochondria respiration stimulated by complex I or complex II substrates by mean of the polarographic method of Chance and Williams [17] using a Clark oxygen electrode, with an oxygraph (Oroboros Oxygraph 2-K, Oroboros Instrument Corp, Graz, Austria). Freshly isolated mitochondria were suspended in 2 mL respiration buffer (100 mM KCl, 1 mM EGTA, 5 mM KH_2_PO_4_, MgCl_2_ 1.2 mM, Tris HCL, pH 7.40 with 1 mg/mL BSA) and incubated at temperatures of interest (6, 10, 15, 20, 25, 31, or 37 °C) in the oxygraph cuvette. Temperature-dependency of complex I and II was assessed by pairs of activator/inhibitor, one after the other on the same mitochondria sample as followed:

- First, glutamate, malate, and pyruvate (GluMP; 20 µL, i.e., 5 mM final concentration in the cuvette for each) was added as electron donors to complex I and to spark the citric acid cycle (CAC). This basal state of oxygen consumption is named state 2.
- After stabilization, the whole mitochondrial respiratory chain (MRC) was started using adenosine diphosphate (ADP) in excess (20 µL, i.e., 2 mM), reaching a maximum activity, called state 3. Then, rotenone (10 µL, i.e., 12.5 µM) was used to inhibit complex I (or more precisely to block electrons transfer to ubiquinone), leading to state 4 [18].
- MRC complex II was then stimulated with succinate (20 µL, i.e., 5 mM) and then inhibited by thenoyltrifluoroacetone (10 µL i.e., 80 µM) [19].

### 2.5. Mitochondrial ROS (H_2_O_2_) Production Rate

H_2_O_2_ released from isolated mitochondria was detected by N-acetyl-3,7-di-hydroxy-phenoxazine assay (Amplex Red, Molecular Probes, Eugen, OR, USA) combined with horseradish peroxidase. Horseradish peroxidase catalyzes reaction between H_2_O_2_ and Amplex Red, generating resorufin on an equimolar basis, which, when excited at 530 nm, emits light at 590 nm. Cold effect on this enzymatic reaction was assessed in a control experiment and no significant variation could be detected at low H_2_O_2_ concentrations (Supplemental Data Figure S2). One could note that 1 pM H_2_O_2_ induced a fluorescent signal on par with the values obtained in mitochondria. Measurement of ROS production was performed using a spectro-fluoro-photometer (F-2500 or F-7000 Digilab, Hitachi, Tokyo, Japan) equipped with thermostatic control. For experiments performed on the F-2500 spectro-fluoro-photometer, a correction factor (multiplication by 2.83, determined by the translation factor between the two standard curves) was applied in order to adjust a similar basal fluorescence value as on the F-7000 at the beginning of the experiment. Fluorescence was measured with peroxidase (20 µL, i.e., 7.4 mU/L) and Amplex red (20 µL, i.e., 2.5 µM), in 2 mL of ROS buffer (EGTA 1 mM, EDTA 1 mM, BSA 1.5 g/L, MgCl_2_ 1.2 mM, Tris 20 mM, pH 7.4) in a spectrophotometer cuvette incubated at the temperature of interest (6, 10, 15, 20, 25, 31, or 37 °C). Following a 2-minute-incubation period at a temperature of interest, mitochondria were added, prior to the substrates 2 min later (GluMP or succinate; 20 µL, i.e., 5 mM each) and ADP (4 µL, i.e., 40 µM). Rotenone (10 µL, i.e., 12.5 µM) was finally added after another 2 min delay. This addition of Rotenone was used to block the electron transport chain within complex I in both forward and reverse mode. Thus, rotenone added after GluMP led to full ROS production by complex I, while rotenone added after succinate blocked RET and forced FET from complex II, thus improving the respiration stimulated by complex II.

ROS assays according to increasing succinate concentrations at different temperatures (15, 20, 25, 31, or 37 °C) were performed with the same procedure with GluMP as initial substrate followed by 10 µL succinate pulses every 1 min and 30 s (10 µL, i.e., an increase in succinate concentration of 1 mM in the cuvette after each pulse). For each temperature, the EC50 of succinate concentrations were determined using a four-parameter logistic regression curve. At 15 °C the ROS production was too low to measure satisfactorily the changes induced by succinate. Data obtained at this temperature were not analyzed.

### 2.6. Mitochondrial Ca^2+^ Retention Capacity

Measurement was performed on spectro-fluoro-photometers (F-2500 or F-7000 Digilab, Hitachi, Tokyo, Japan) equipped with thermostatic control. For some experiments performed, a standardization factor (multiplication by 2) was applied on fluorescence values in order to obtain the same basal fluorescence value for all experiments. Extra-mitochondrial Ca^2+^ concentration was recorded using the Ca^2+^ sensitive probe Ca^2+^ green-5N (0.5 µM) (Molecular Probes, Eugene, OR, USA) with excitation and emission wavelengths set at 500 and 530 nm, respectively.

CRC was determined at temperatures of interest (15, 20, 25, 31, and 37 °C). Experiments were not performed below 15 °C because of extremely low Ca^2+^ absorption under this temperature. Briefly, fresh mitochondria were suspended in 2 mL buffer (150 mM sucrose, 50 mM KCl, 2 mM KH_2_PO_4_, in 20 mM Tris/HCl, pH 7.40) in a spectrophotometer cuvette, with specific substrates in order to spark mitochondrial respiration and Krebs cycle, namely, glutamate (5 mM), malate (5 mM) or succinate (concentration was set at ROS production EC50 for each temperature), and MgCl_2_ (1.2 mM) and ADP (40 µM). Contrary to the other experiments, pyruvate was not added because we found that it inhibited Ca^2+^ absorption by mitochondria, as demonstrated in the control experiment (Supplemental Data Figure S3A). We also controlled that GM in addition of succinate did not prevent Ca uptake (Supplemental Data Figure S3B).

After the absorption of the previous pulse was complete (as observed by the plateau phase in the decline of fluorescence), 10 nmol CaCl_2_ pulses were performed. The amount of CaCl_2_ necessary to trigger a massive Ca^2+^ release, corresponding to mPTP opening, was used as an indicator of mitochondrial maximum CRC.

CRC was calculated as previously described [20] (sum of amount of Ca^2+^ added until opening of the mPTP) and with a calibration-based method (Supplemental Data Figure S3C). We observed that basal fluorescence of Ca^2+^ green-5N was lower in presence of mitochondria (even when mitochondria were uncoupled with addition of FCCP) and that this decrease was proportional to the mitochondria amount. In order to take in account this quenching of fluorescence, we subtracted the basal fluorescence level in all our calculation prior to figure out the real Ca^2+^ amount absorbed by mitochondria as followed:

- Equation (1) is given by the fit of the calibration curve

(1) $$\;F=B+\frac{\left(T-B\right)}{1+10^{\left(logEC50-x\right)*H}}$$

where *F* is the fluorescence intensity, *B* is the bottom value of the calibration curve, *T* is the top value of the calibration curve, LogEC50 is the logarithm of the [Ca^2+^] at which 50% of the probe is bound, *x* is the log(amount of Ca^2+^) in the cuvette in nmol and *H* is the hill slope of the calibration curve.

- Ca^2+^ concentration in the cuvette could be determined back with the fluorescence value using the transformed Equation (1) into Equation (2)

(2) $$\;x=\log\left(EC50\right)-\frac{log\left(\frac{T-B}{F-B}-1\right)}{H}$$

where *F* is the fluorescence intensity after subtraction of the fluorescence noise (determined as the average fluorescence during the first 1 min of recording; F0) or by the averaged fluorescence before a Ca^2+^ pulse when its level was below F0. Thus, CRC was calculated as the difference between the total amount of Ca^2+^ added in the cuvette (number of pulses multiplied by the amount of Ca^2+^ in each pulse) and the remaining amount of Ca^2+^ in the cuvette just before mPTP opening.

Ca^2+^ uptake rate and Ca^2+^ concentration equilibrium were assessed using the second pulse of the Ca^2+^ absorption curve, after normalization to the maximum peak fluorescence value. The second absorption curve was chosen because in heart, the first curve had, usually, a different profile from others absorption curves in heart or in brain. Ca^2+^ uptake rates and Ca^2+^ equilibrium concentrations were calculated according to a one phase exponential decay regression model. Experiments were performed with, and without, a mitochondrial NCLX inhibitor (CGP-37157) at a concentration of 20 µM. Indeed, the NCLX drives the efflux of Ca^2+^ from the mitochondrion, whereas the MCU drives the influx [21].

### 2.7. Mitochondrial Membrane Potential (Ψm)

Tetramethylrhodamine, Methyl Ester, Perchlorate (TMRM, ThermoFisher, Waltham, MA, USA) assay was performed on spectro-fluoro-photometers (F-2500 or F-7000 Digilab, Hitachi, Tokyo, Japan; λex 550 nm, λem 580 nm) equipped with thermostatic control. TMRM fluorescence was measured in the bath, therefore a decrease in fluorescence is associated with the hyperpolarization in Ψm while an increase in fluorescence reports a depolarization in Ψm. Briefly, TMRM was used at 20 nM for about 120 s, then substrates were loaded: (5 mM), malate (5 mM) or succinate (concentration was set at ROS production EC50 for each temperature) in order to make mitochondria respire at the basal level (state 2). After the equilibrium in Ψm was reached, MgCl_2_ (1.2 mM) and ADP (40 µM) were added to induced ATP synthesis-driving respiration for about 120 s. Finally, 10 µM FCCP was added to uncouple respiration and ATP synthesis that led to disruption of Ψm. After correction of the cold-dependent effect on TMRM, the depolarization induced by state 2 was estimated as the normalization of the mean fluorescence after FCCP by the mean fluorescence after substrates addition. The depolarization induced during state 3 respiration was estimated as the normalization of the mean fluorescence after FCCP by the mean fluorescence after ADP + MgCl_2_.

### 2.8. Chemicals

Unless otherwise specified, chemicals were purchased from Sigma Chemical (Sigma-Aldrich, St. Louis, MO, USA).

### 2.9. Statistics

Values were expressed as mean ± 95% confidence interval (IC95%) to measure the reproducibility of data values and mean ± SEM when the precision of the mean value was important for the interpretation of data. To assess temperature effect, Q_10_ was used: it is a measure of the rate of change in a biological or chemical system with increasing the temperature by 10 °C. Q_10_ value was determine with the following Equation (3):

(3) $$Q_{10}={\left(\frac{k_{T2}}{k_{T1}}\right)}^{\frac{10}{\left(T2-T1\right)}}$$

Calculation of Ca^2+^ uptake rate by mitochondria was modelized by a one phase decay fit from the peak of fluorescence triggered by the second Ca^2+^ pulse. The rate constant, K (s^−1^), was extracted for all fits. Data were analyzed using GraphPad Prism 8.4.1 software (GraphPad Software, La Jolla, CA, USA). All reported *p* values are two-sided, and a *p* < 0.05 was considered statistically significant.

## 3. Results

### 3.1. Effect of Cold from 6 to 37 °C on Mitochondrial Oxygen Consumption Rate

**Figure 1:**
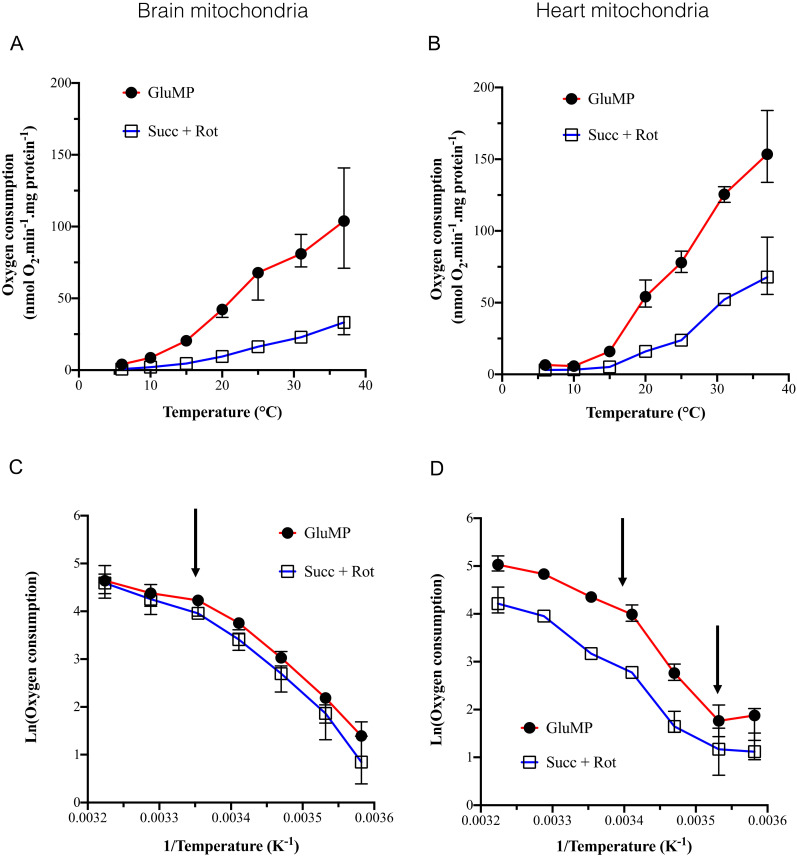
The dependance of mitochondrial oxygen consumption on the temperature. (**A**) Mitochondrial oxygen consumption in brain mitochondria, in nmol O_2_/min/mg protein, according to temperature (6, 10, 15, 20, 25, 31, and 37 °C). For each temperature, each experiment was performed on the same sample in the same oxygraph cuvette: GluMP was first added, followed by rotenone and succinate. *n* = 4 to 7 for each temperature. Data are expressed as mean ± IC95%. (**B**) as A for heart mitochondria. (**C**,**D**) Arrhenius plots of data from (**A**,**B**) panels, respectively, expressed as % of maximum. Mean ± IC95%. Arrows indicate characteristic inflexion point in the activation energy.

To assess the influence of cold, isolated brain and heart mitochondria (BM and HM) oxygen consumption was measured at different temperatures from 37 to 6 °C with an interval of 5–6 °C. To assess difference in the cold-dependency of complex I and II, we compared both Glutamate/Malate/Pyruvate- (GluMP) and succinate- (Succ) driven respiration (Figure 1).

Addition of rotenone to succinate prevented RET towards complex I, making possible to assess the complex II-driven oxygen consumption. Oxygen consumption was inhibited by 90% between 37 and 15 °C in both BM and HM. Between 37 and 6 °C (*p* < 0.001) with the temperature coefficient, Q_10_, was 2.76 and 2.83 in the presence of GluMP in BM and HM (Table 1), respectively, and 3.25 and 2.71 in presence of Succ + rotenone in BM and HM, respectively (Table 1). These Q_10_ values are similar to reported ones within the range of Arrhenius effect on mesophilic and thermophilic enzymes and they correspond to a low temperature sensitivity [22,23]. Along the line of previous studies [12,13], Arrhenius plots revealed inflexion points in the activation energy (Ea) around 25 °C in both BM and HM but also at 10 °C only in HM (Figure 1C,D). Below 10 °C, respiration was almost abolished supporting the fact that deep cold suppresses metabolic activity in both BM and HM (Figure 1A,B). At high temperature, respiration was not significantly modified between 37 and 31 °C. Within the range of 25 to 6 °C in BM, Q_10_ raised to around four independently of the substrate (Table 1), which supported the involvement of the general denaturing effect of cold on enzymes. In HM, the strongest cold-dependence was found within the range of 25–10 °C in the presence of GluMP with a Q_10_ value of 9.21 (Table 1). Finally, when we normalized the respiration rate by the content of live mitochondria, they were almost similar in both BM and HM samples at each temperature excepted a higher Succ-driven respiration in HM at 31 °C and 37 °C (Supplemental Data Figure S4A).

### 3.2. Effect of Cold from 6 to 37 °C on ROS Production Rate in Energized Mitochondria

**Figure 2:**
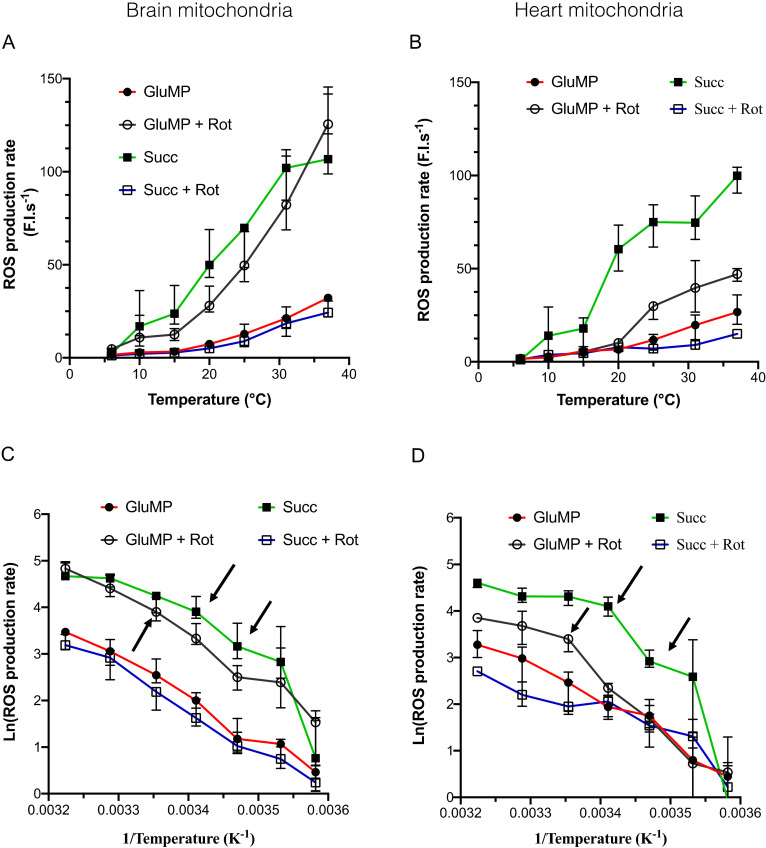
The dependance of mitochondrial ROS production rate on the temperature. (**A**) ROS production rate in brain mitochondria, in arbitrary fluorescence unit/sec, according to temperature (6, 10, 15, 20, 25, 31, and 37 °C). For each temperature, each experiment was performed on the same sample in the same cuvette: GluMP (respectively, succinate) was first added, followed by rotenone. *n* = 4 to 7 for each temperature. Data are expressed as mean ± IC95%. (**B**) as A for heart mitochondria. (**C**,**D**) Arrhenius plots of data from (**A**,**B**) panels, respectively, expressed as % of maximum. Mean ± IC95%. Arrows indicate characteristic inflexion point in the activation energy.

In normoxic condition, ROS are generated as byproducts of the electron transport chain (ETC) activity. We assessed whether cold effect on ETC-mediated ROS production decreased along with temperature. In BM (Figure 2A), GluMP substrates induced a basal ROS production rate, which was enhanced after the addition of rotenone blocked electron transfer from complex I to ubiquinone (CoQ) and induced, as expected, massive ROS production by complex I.

Interestingly, this ROS production rate was similar to the one induced by reverse electron transfer (RET), as shown when succinate is added alone as a substrate. After adding rotenone to succinate, ROS production rate fell to the basal level similar to the one induced by GluMP, one order of magnitude between complex I-mediated maximal ROS production. Surprisingly, in HM, GluMP and rotenone treatment induced a significantly lower ROS production rate than the one induced by succinate (Figure 2B). This suggests that RET-driven ROS production at complex I ubiquinone-binding site (I_Q_ site) is more efficient that NADH Dehydrogenase-driven ROS production at complex I flavin site (I_F_ site) in HM [24]. The highest drop in ROS production rate was seen between 31 and 15 °C in both organs and for both substrates, with a decrease of almost 85%, similarly to the decrease in the respiration rate in the same range of temperatures.

Arrhenius plots of the ROS production rate were used to reveal its dependence on cold, and compared the effect of the substrates. In both BM and HM, temperature-sensitivity was almost similar in each of the substrate conditions with a Q_10_ value around three between 37 and 6 °C (Figure 2C,D; Table 1). Inflexion points in the Arrhenius plot were 25–20 °C, for GluMP and Succ, respectively; 15 °C—because of the greater uncertainty in the measures at 10 °C, we did not take it into consideration. In both organs, a strong cold-dependence of ROS production was found for the Succ condition in the range (6–15 °C) with Q_10_ values above 10. Such high cold-dependence was not observed for respiration, which suggests a specificity in the reaction chain leading to ROS production by RET—likely a cold-dependence at the complex I I_Q_ site. This effect was also visible between 25 and 15 °C in HM (Q_10_ = 15.58 and 4.11 in HM and BM, respectively), which suggests a difference in cold-sensitivity of RET-mediated ROS production in HM. ROS production at the complex I I_F_ site (GluMP + rotenone condition) showed a lesser cold-dependency than at the I_Q_ site: Q_10_ = 3.2 in BM between 15 and 6 °C and Q_10_ around five in HM between 25 and 6 °C. In addition, these temperature coefficients were similar to the ones observed for respiration rate, which implies a common mechanism. Finally, we normalized the ROS production rate by the content of live mitochondria in both BM and HM samples at each temperature (Supplemental Data Figure S4B). The ROS production rate was found to be higher in BM than in HM, but no differences could be seen between RET-induced ROS production and the GluMP-induced one in BM. Conversely in HM, RET-driven ROS production was more efficient than the GluMP-induced one, which suggests a more preeminent role of the complex I I_Q_ site in ROS production than the I_F_ site.

### 3.3. Effect of a Mixed Substrate on ROS Production by Complex I and Its Sensitivity to Cold

**Figure 3:**
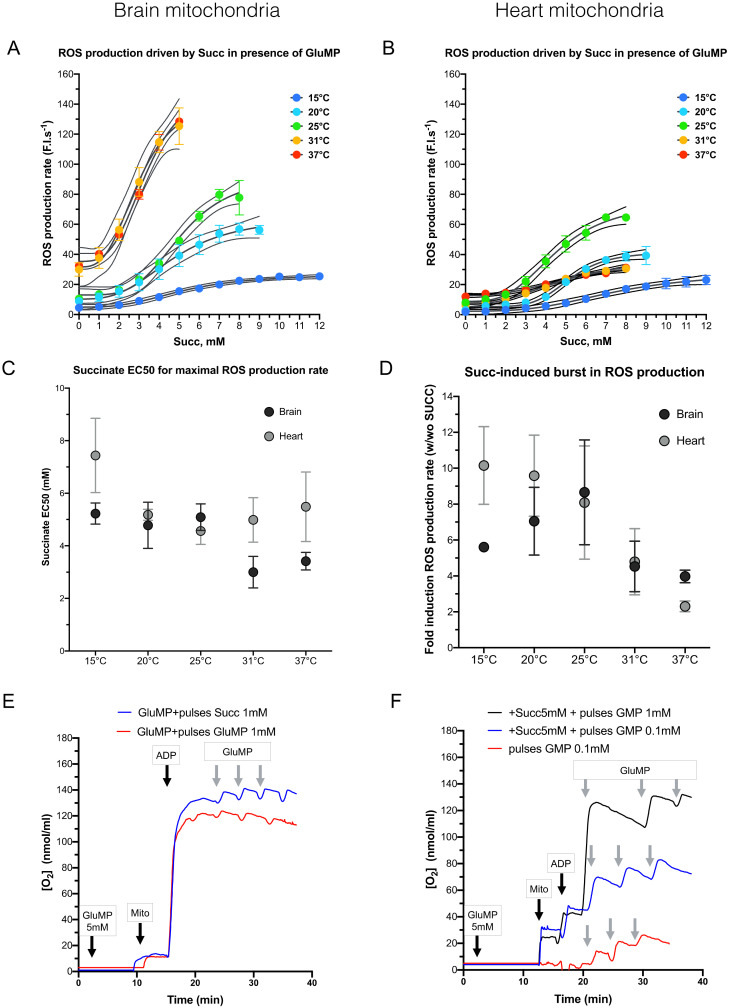
The dependance of mitochondrial ROS production rate on increasing succinate concentrations. (**A**) ROS production rate in brain mitochondria, in arbitrary fluorescence unit/s (F.I.s^−1^), according to increasing succinate concentrations at different temperature (15, 20, 25, 31, and 37 °C). For each temperature, each experiment was performed on the same sample in the same cuvette: GluMP was first added, followed by increased amount of succinate. *n* = 3 for each temperature. Data are expressed as mean ± SEM. Dotted lines represent logistic regression IC95%. (**B**) as A for heart mitochondria. (**C**). Succinate concentration EC50 for maximal ROS production rate at each temperature, extracted from the logistic regression shown in the panels (**A**,**B**). Data are expressed as mean ± SEM. (**D**). Histogram represents Succ-induced burst in ROS production. The values were calculated by divided the ratio of ROS production rate calculated in presence of Succ (w) over its value in absence of Succ (wo). Data are expressed as mean ± IC95%; *n* = 3 for brain mitochondria and =4 for heart mitochondria. (**E**). Representative time trace of O_2_ consumption in mitochondria fed with 5 mM GluMP. Addition of unlimited ADP induced complex V-induced ETC activity. Gradual supplementation of either 1 m Succ or 1 mM GluMP did not significantly modified the respiration rate. (**F**). Representative time trace of O_2_ consumption in mitochondria fed with 5 mM Succ. Addition of unlimited ADP was not sufficient to induce significantly complex V-induced ETC activity. Gradual supplementation of either 0.1 mM or 1 mM GluMP significantly increased respiration until it reached maximal activity as in panel E. Absence of the initial 5 mM Succ (red line) did not prevent respiration induction when 0.1 mM GluMP was added.

Succinate accumulation during ischemia is thought to trigger a ROS burst through enhancement of RET at the onset of reperfusion [7]. This theory infers that RET can overpower FET in some specific conditions. However, an interesting alternative could involve the over-reduction in the ubiquinone (CoQ) pool through electrons coming from both complex I and II [8]. Over-reduced CoQ would, thus, create a bottleneck for the electron pass that would increase an electron leak from complex I via ROS production. We thus tested whether the RET-outperforming FET hypothesis or the CoQ over-reduction one promoted ROS production in normoxia, and we figured out its cold-sensitivity at the same time. Experimentally, we measured the ROS production rate in respiring condition: 5 mM GluMP + Mg^2+^ + ADP. Then, 1 mM Succ pulses were periodically added until it reached 10 mM. ROS production rate was quantified for each interval. These experiments were performed within the range (37–15 °C) which, we showed, was associated with an 85–90% drop in both respiration and ROS production in both BM and HM. As shown in Figure 3A,B, an increase in Succ concentration enhanced ROS production at all temperatures. However, sensitization to Succ was not equal at each temperature in the mitochondria of the two organs. Half maximal effective succinate concentration (EC50) for ROS production rate was extracted with a sigmoidal fit (Figure 3C) and compared with each other at all temperatures: 5.2 ± 0.4, 4.8 ± 0.9, 5.1 ± 0.5, 3.0 ± 0.6, and 3.4 ± 0.3 mM, for 15, 20, 25, 31, and 37 °C, respectively, in BM, with a significant difference between 15 and 31 °C (*p* = 0.033) and almost significant between 25 and 31 °C (*p* = 0.052). In HM, Succ EC50 were: 7.4 ± 1.4, 5.2 ± 0.2, 4.6 ± 0.5, 5.0 ± 0.8, and 5.5 ± 1.3 mM for 15, 20, 25, 31, and 37 °C, respectively, with a significant difference between 15 and all other temperatures (*p* = 0.008, 0.0006, 0.004, and 0.031 for 20, 25, 31, and 37 °C, respectively). Along the line of the results presented in the Figure 2, this increase in Succ EC50 with deeper cold conditions suggests that mitochondria are more resistant to increased Succ concentration and, consequently, less prone to ROS production at physiological Succ concentrations. No difference in the ROS production rate and in Succ EC50 could be seen between 31 and 37 °C. Along the line of Kohlhauer et al., these results confirm that the protective effect of mild therapeutic hypothermia around 32 °C is independent of succinate-driven ROS production in BM and HM [25].

Although ROS production rate is maximized at the highest Succ concentration above 31 °C in BM and at 25 °C in HM (Figure 3A,B), the fold-induction caused by high Succ concentration is maximized at 25 °C in both organs (Figure 3D). This implies that 25 °C is possibly the worst cold temperature to reduce ROS production. Noteworthily, 25 °C is also the temperature at which ROS production rate is maximized in HM (Figure 3B). Although ROS production by RET (Succ alone; Figure 2A) was similar to that of ROS production in mixt substrate condition in BM at any temperature (Figure 3A,B), ROS production in mixt substrate condition was threefold lower than that of ROS production by RET (Succ alone) between 37 and 31 °C.

Involvement of complex I I_F_ site in BM could implicate a RET phenomenon. To test this hypothesis, we hypothesized that a competition between RET and FET would lead to a decrease in the net FET flux, thus causing a decrease in respiration rate when Succ concentration increase. As shown in the Figure 3E,F, neither gradual addition of mM Succ concentration on GluMP-fueled mitochondria, nor gradual addition of mM GluMP concentration on Succ-fueled mitochondria at 25 °C did modify the respiration rate, which was maintained at its maximal activity. This confirms that RET did not occur in mixt substrate condition but that the boost of ROS production caused by gradual addition of mM Succ concentration on GluMP-fueled mitochondria likely relies on over-reduction in the CoQ pool.

### 3.4. Effect of temperature from 15 to 37 °C on Ca^2+^ retention capacity in energized respiring mitochondria

Ca^2+^ retention capacity (CRC) is assessing the potency of mitochondria to uptake Ca^2+^ before the opening of mPTP. We measured CRC at 15, 20, 25, 31, and 37 °C in mitochondria energized by Mg^2+^-ADP and fueled by GluM and succinate in order to fit the most physiological condition possible. Pyruvate was removed from the solution since we found out that it perturbates Ca^2+^ measurement through quenching the fluorescence of Calcium Green 5N (Supplemental Data Figure S3A). We demonstrated in the Figure 3 that Succ had a differential enhancing effect on ROS production within the range of temperature (15–37 °C), and ROS are thought to increase the susceptibility of the mPTP opening [20]. In order to neutralize a possible cofounding effect of ROS on the mPTP opening, we measured the CRC value at the succinate EC50 for ROS production. Mixt substrate slowed down drastically the Ca^2+^ uptake rate by mitochondria, compared to Succ alone condition, likely due to the respiratory level and mitochondrial membrane potential (Supplemental Data Figure S3B). Since Ca^2+^ uptake after each pulse of Ca^2+^ was incompletely up taken by HM, calculation of the CRC value by multiplying the number of Ca^2+^ pulses by the nmoles of Ca^2+^ in each pulse was biased.

**Figure 4:**
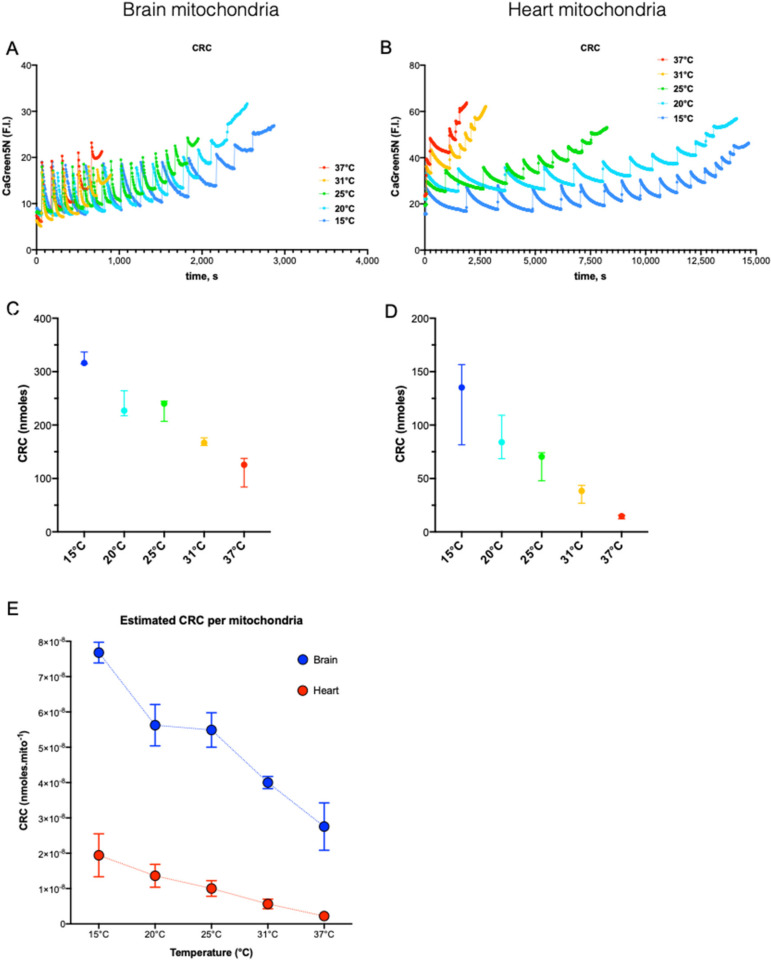
The dependance of Ca^2+^ retention capacity on the temperature. Example of Ca^2+^ retention capacity (CRC) traces for brain (**A**) and heart (**B**) at different temperatures. (**C**,**D**). CRC in nmol of Ca^2+^ calculated by the calibration curve method at different temperature (15, 20, 25, 31, and 37 °C). *n* = 3 for each temperature. Data are expressed as median ± 95%CI. (**E**). CRC value normalized by the estimated content of live mitochondria in each brain and heart mitochondria samples.

In both organs, cold increased CRC of mitochondria from 37 to 15 °C (Figure 4C,D). This inverse correlation between temperature and CRC was almost linear with 8.7 and 4.9 additional nmoles of Ca^2+^ uptaken per 1 °C decrease in brain and heart mitochondria, respectively (Supplemental Data Figure S5C,D; R^2^: 0.90 and R^2^: 0.82 (*p* < 0.001 for both). Corrected by the number of live mitochondria estimated by flow cytometry (Supplemental Data Figure S1 and Section 2), BM were found to accumulate 4 and 12 time more Ca^2+^ than HM at 15 and 37 °C, respectively (Figure 4E). Finally, we observed that CRC did not rise linearly with cooling but showed a transient plateau between 20 and 25 °C. Lee et al. reported previously this particularity on the Arrhenius plot of an indirect measurement of Ca^2+^ uptake in depolarized mitochondria (inhibited by both rotenone and antimycin) [13]. Besides the cold-dependence of Ca^2+^ uptake, cold-dependence of NCLX and indirect estimates of the cold-dependence of MCU have been studied in permeabilized Hela cells at 22and 37 °C with a mixed substrate condition [26].

We thus calibrated the Calcium Green 5N dose–response curve on the two spectrophotometers at all temperatures (Supplemental Data Figure S3C) in order to estimate the real amount of Ca^2+^, as extensively described in the method section. In BM, we found that CRC estimated by the pulse count method was giving similar results to that of the calculated methods (Supplemental Data Figure S5A), which was expected by the almost complete Ca^2+^ uptake after each pulse (Figure 4A). These results also validated the precision of our method. In HM, strong differences were found between the two methods and the lower CRC was, the higher was the error made by the pulse count method (Supplemental Data Figure S5B). As shown in the Figure 4B, Ca^2+^ uptake in HM was largely uncomplete at 37 °C, but far more efficient at 15 °C, which may also explain why the error given by the pulse count method was greater at 37 °C. Finally, we normalized CRC by the count of live mitochondria and we found that HM had a lower unitary CRC than BM (Figure 4E) but that this difference decreases while cooling (12-fold at 37 °C and 4-fold at 15 °C). We wondered if the cold-decency of the Cyclosporine (CsA)-mediated inhibition of mPTP could explain this effect but we did not detect interplay between cold and CsA (Figure S5C).

### 3.5. Effect of Temperature from 15 to 37 °C on Ca^2+^ Dynamics across the Mitochondrial Membranes in Energized Respiring Mitochondria

**Figure 5:**
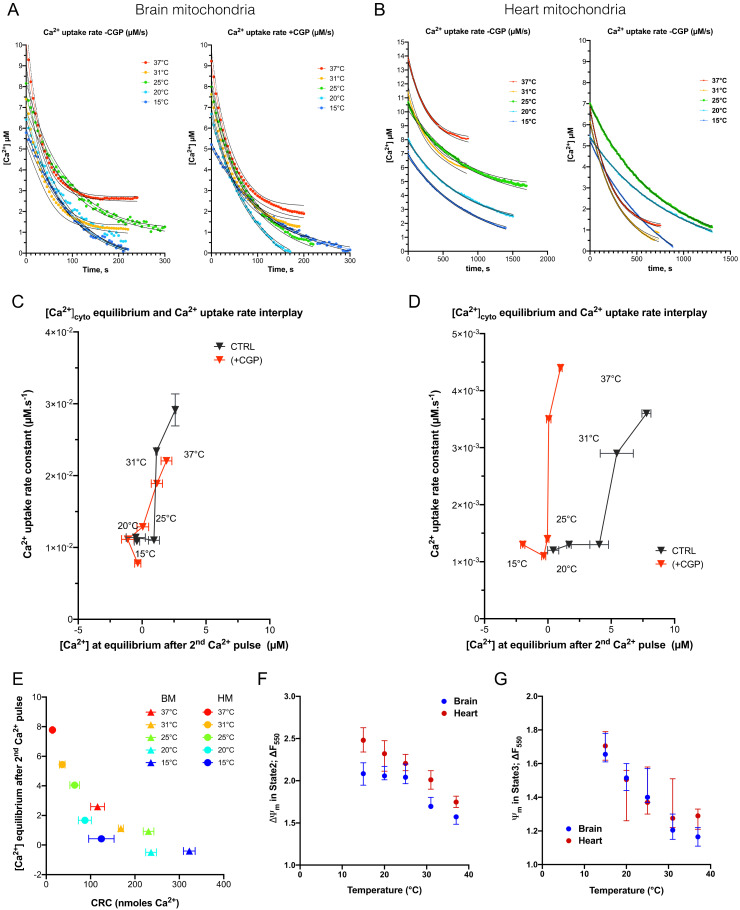
The dependance of Ca^2+^ uptake rate and steady-state [Ca^2+^] concentration in mitochondria on the temperature. (**A**), [Ca^2+^] over time at each temperature presented as the Calcium Green 5N fluorescence decay after the second pulse of Ca^2+^ of each CRC assay. Values were normalized by the peak value at origin of each curve. Experiments conducted with or without NCLX inhibitor (CGP). Dotted lines represent logistic regression IC95%. (**B**), as A for heart mitochondria. (**C**,**D**), biplots represent interplay between [Ca^2+^] at equilibrium and Ca^2+^ uptake rate in mitochondria of brain and heart, respectively. Data were extracted from Figure 5A,B. Data are expressed as mean ± SEM (**E**)*,* Correlation between steady-state [Ca^2+^] concentration and CRC values in BM and HM. Ψm estimation was figured out in either state 2 respiration (**F**) or state 3 respiration (**G**) in both brain and heart mitochondria at different temperatures as reported in the M and M Section.

We observed strong changes in Ca^2+^ dynamics in our CRC experiments and we further analyzed the cold-dependency of the Ca^2+^ uptake rate and Ca^2+^ equilibrium outside of brain and of heart mitochondria. Because we observed that the dynamic of Ca^2+^ uptake after the first Ca^2+^ pulse was more heterogenous from one experiment to another, we have chosen to analyze the dynamic of Ca^2+^ uptake after the second Ca^2+^ pulse. [Ca^2+^] was figured out by mean of calibration curves from the peak value measured at the onset of the Ca^2+^ pulse and an exponential decay was fitted on averaged values at each temperature (Figure 5A,B). The exponential decay was used to figure out the Ca^2+^ uptake rate while the Ca^2+^ concentration out-of-mitochondria at equilibrium was extrapolated from the bottom value of the fit. Ca^2+^ uptake rate is determined by the true Ca^2+^ uptake by the mitochondrial Ca^2+^ uniporter, MCU (confirmed by addition of 1 µM RU360, a MCU inhibitor, which prevents Ca^2+^ entry in the mitochondria as reported in the Supplemental Figure S5C), which is counterbalanced by Ca^2+^ extrusion by the mitochondrial Na^+^/Ca^2+^ exchanger, NCLX. This latter was prevented by treatment of mitochondria with 20 µM of CGP-37157 in order to better appreciate the cold-dependence on Ca^2+^ uptake rate (Figure 5A,B). Plotting both the Ca^2+^ uptake rate and the Ca^2+^ equilibrium concentration helps us understand that NCLX inhibition had a major effect on the Ca^2+^ equilibrium concentration outside of HM (*p* < 0.005), as observed in the Figure 5C,D and the Supplemental Figure S5E. Cold also had an inhibitory effect on the Ca^2+^ equilibrium concentration outside of HM, which was suppressed in presence of CGP-37157. This suggests that cold-dependence of NCLX plays an important role on the regulation of Ca^2+^ equilibrium concentration and is, noteworthily, linearly correlated to the increase in CRC values in HM (Figure 5E). Besides this role, NCLX inhibition was accompanied by a reduction in the Ca^2+^ uptake rate in both BM and HM at 37 °C.

In BM, Ca^2+^ uptake rate decreased significantly while temperature drops from 37 to 25 °C: 2.91 × 10^−2^ µM.s^−1^ ((2,53 × 10^−2^; 3.43 × 10^−2^) 95% CI) to 1.10 × 10^−2^ µM.s^−1^ ((0.77 × 10^−2^; 1.22 × 10^−2^) 95% CI) (*p* = 0.009). In HM, the Ca^2+^ uptake rate decreased significantly between 37 and 25 °C: 3.63 × 10^−3^ µM.s^−1^ ((3.13 × 10^−3^; 4.18 × 10^−3^) 95% CI) to 1.35 × 10^−3^ µM.s^−1^ ((1.06 × 10^−3^; 1.64 × 10^−3^) 95% CI) (*p* < 0.0001) (Figure 5D). These values and the fold repression by cold were both of the same order of magnitude as the one reported in a model of rabbit heart mitochondria [27]. These cold-induced decreases in Ca^2+^ uptake rate were maintained in the presence of CGP-37157, which suggests that NCLX-mediated Ca^2+^ extrusion was not involved. The Arrhenius plot of the Ca^2+^ uptake rate confirmed that cold-dependence of the Ca^2+^ uptake rate lay between 37 and 25 °C (with a maximal activation energy between 31 and 25 °C) and was almost insensitive to CGP-37157 treatment (Supplemental Figure S5F,G).

Since the decrease in the Ca^2+^ uptake rate at the lower temperatures could have been caused by Ψm depolarization, we estimated Ψm in all these conditions by measuring changes in TMRM fluorescence. We first detected that TMRM fluorescence was linearly sensitive to cooling (Supplemental Figure S6A) and, thus, could be corrected (Supplemental Figure S6B) prior to analyzing changes in Ψm over the experiments (Supplemental Figure S6C,D). We found that Ψm hyperpolarization increased when the temperature decreased, independently of ATP synthase activity (Figure 5F). Indeed, both state 2 and state 3 of respiration were associated with the increased polarization of Ψm (Figure 5G). These results demonstrate that the electrochemical gradient for Ca^2+^ uptake in mitochondria was maximum in cold conditions. Therefore, the decrease in the Ca^2+^ uptake rate when cooling the bath was not related to a drop in Ψm. All together, these data support the fact that the cold-mediated inhibition of Ca^2+^ uptake rate is due to inactivation of MCU activity mainly in the range (31–25 °C) in both brain (Q_10_: 3.32) and heart (Q_10_:3.81) (Table 1).

## 4. Discussion

Mitochondrion is at the crossroad of many cellular processes and it plays a key role in cellular death following I/R injury. Cold is an important factor in protecting against I/R injury. Surprisingly, only a few studies have explored the relationship between mitochondrial functions and cold. In our study, we explored the cold-sensitivity of respiration, ROS production, and CRC in both brain and heart mitochondria in normoxic conditions.

We confirmed the previously reported cold-dependence of respiration with a major breaking point in its activation energy around 25 °C [12,13,28]. However, contrarily to these studies, we showed that (i) the cold-dependence of respiration was equivalent to whatever the substrate (GluMP or Succ + rotenone); (ii) the break point in activation energy of BM was 25 °C but was around 20 °C in HM. Two studies addressed this topic through a wide range of temperatures (37–7 °C) in mitochondria of homeothermic animals, fueled with glutamate malate and ADP-Mg^2+^ [28,29]. They found a similar trend with a strong inhibition of respiration below 25 °C and Q_10_ between 2 and 4. Another study on brain squirrel mitochondria [30], in a narrower range of 37 to 28 °C, found a decrease of around 60% in mitochondrial GluMP or succinate-driven respiration. In mild cold condition (31 °C), the inhibition of respiration was 20%, but fell to an 80% decrease at 15 °C. The maximal cold-sensitivity was found between 25 and 6 °C that suggests that the cold-dependence could likely be related to protein denaturation happening gradually below 25–20 °C. Lemieux et al. reported that succinate addition to GluMP-fueled mitochondria enhanced their respiration but did not modify the cold sensitivity (Q_10_ ~ 4 between 25 and 4 °C) [28]. Our data confirmed that addition of Succ to GluMP could enhance respiration, and at least did not impair it (Figure 3E,F). Lemieux et al. also concluded that the NADH-dependent respiration was enhanced while the temperature decreased. In our study, Q_10_ was around four in BM but reached nine in HM fed with GluMP while it dropped to three in Succ-fed HM in the range (20–10 °C). Despite the Q_10_ estimates, which can suffer from the divergency between the exponential models used [22,31], the Arrhenius plots did not show a lesser decrease in respiration in GluMP-fueled HM than in Succ-fueled ones (Figure 1C,D).

ROS are mainly produced by mitochondria, as a byproduct of the MRC, in normoxia, as well as in the I/R setting. A two-site model of ROS production at complex I was proposed ten years ago [32]. One site (I_F_) is supposed to be at the NADH-binding site, the other one (I_Q_), at the ubiquinone-binding site. The first is thought to be implied in ROS production during FET, the second during RET. We explored these two sites’ cold dependency, I_F_ alone in GluMP-fueled mitochondria incubated with rotenone, and I_Q_ with Succ alone-fueled ones. In mild cold condition (31 °C), the I_Q_-mediated ROS production was decreased by 15%, but fell to an 85% decrease at 15 °C in BM and HM. I_F_-mediated ROS production was decreased by 15% in HM but 45% in BM, and fell to a 90% decrease at 15 °C in HM. This difference in I_F_-mediated ROS between BM and HM is supported by a difference in the activation energy, which decreased linearly with cooling in BM but started to decrease below 25 °C in HM (Figure 2C,D). These results suggest that the composition (in sub-units or partner proteins) of complex I must be different in BM and HM in order to explain this difference.

In both BM as in HM, I_Q_-mediated ROS is relatively stable in the range (37–20 °C), which means that while respiration decreases, the potency of RET to produce ROS is maintained. Below 15 °C, I_Q_-mediated ROS cold-sensitive increases drastically, which could prevent ROS production through RET. Noteworthily, we found that the ROS production rate in BM was equivalent in both substrates, while ROS production via the complex I I_Q_ site was two times greater than via the complex I I_F_ site in the range (37–20 °C) (Figure 2D and Supplemental Figure S4B). In both organs, the cold-dependence of ROS production was higher (higher Q_10_ values) in substrate conditions maximizing ROS production: GluMP + rot and Succ alone (Table 1). This indicates that the complex I I_F_ site + complex III and complex II + complex III are less sensitive to cold than the pure complex I I_F_ site or complex II + complex I I_Q_ site.

A recent study introduced a novel paradigm in ROS implication in I/R injury physiopathology by showing that the accumulated Succ during ischemia was rapidly oxidized by complex II at the onset of reperfusion, which could lead to massive mitochondrial ROS production through RET at complex I [7]. However, this theory has never accounted for the maintenance of complex I activity during the enhanced complex II stimulation with succinate. As succinate accumulation during in vivo ischemia is proportional to the duration of ischemia [7], we studied mitochondrial ROS production according to increasing succinate concentrations. First of all, we demonstrated that ROS production induced by the addition of Succ required a higher (Succ) than (GluMP) at physiological temperatures. In addition, when temperature decreases, the requirement for Succ increases to boost ROS production in the presence of complex I substrates. This may explain why cold is known to reduce ROS production in cells. This boost in ROS production could indeed be caused by RET as proposed by Chouchani et al. [7], but could also rely on a bottleneck in the electron path caused by the over-reduction in the CoQ pool [8]. Involvement of RET would uncouple substrate consumption and respiration efficiency with a decrease in maximal respiration rate because RET would compete with FET. However, we observed a small increase in respiration what argued for a mechanism, different from RET, in which the respiration rate is already maximized and the electrons overproduced are released through ROS production. This mechanism would involve the over-reduction in the CoQ pool, which limits the ability of electrons produced by NAD Desyhydrogenase to be taken in charge in FET. These electrons will thereby leak through the complex I I_F_ or I_Q_ sites. In our study, BM produced ROS in mixt substrate condition at a maximal rate similar to the one induced by Succ alone or GluMP + rotenone. However, the boost of ROS production induced by addition of Succ in the presence of GluMP is maximized below 25 °C (Figure 3D), a threshold temperature at which respiration decreases, whatever the substrate, ROS production at the complex I site I_F_ site decreases, while ROS production at the complex I site I_Q_ site is maintained. It is, thus, likely that ROS overproduction in mixt substrate condition mainly relies on the complex I site I_Q_ site. Noteworthily, we found that in HM, the boost in the ROS production rate in mixt substrate condition (Figure 3) was equivalent to the ROS production rate driven by Succ alone but within the range (25–15 °C) but similar to the ROS production rate driven by GluMP + rotenone between 31 and 37 °C. This suggest that ROS production in mixt substrate condition is achieved at the complex I I_Q_ site in the range (25–15 °C). However, the decrease in ROS production rate between 31 and 37 °C in HM is likely due to the uncoupling between respiration and ROS production. Indeed, while the respiration rate fall by 50%, the maximal ROS production by the complex I I_F_ and I_Q_ sites only decreased by 39% and 25%, respectively. By contrast, in BM fueled by mixt substrate condition, the respiration rate fall only by 32% when maximal ROS production by the complex I I_F_ and I_Q_ sites only decreased by 58% and 43%, respectively. This uncoupling between respiration and ROS production rates between BM and HM in mixt substrate condition could shed light on the results found in neuroprotection in clinical studies [2,4].

Mitochondrion Ca^2+^ homeostasis plays a key role in bioenergetics and in the protection of the cell by buffering the Ca^2+^ overload in the cytosol. However, mitochondria can absorb Ca^2+^ to a saturation threshold above which mPTP opens and causes a massive detrimental release of Ca^2+^. So, increasing the CRC value could be a relevant aim to protect cells against Ca^2+^ overload at reperfusion. In this study, we found an almost linear relation between CRC and cold, with a plateau in the range (25–20 °C) in BM and a limited increase in HM. Interestingly, BM displayed a higher absolute value of CRC per mitochondria, whereas the difference decreased when cooling down (12-fold at 37 °C to 4-fold at 15 °C). To go further into the characterization of the cold effect on the Ca^2+^ homeostasis dynamic, we measured both Ca^2+^ uptake rate and steady-state [Ca^2+^] outside mitochondria and figured out the role of NCLX on these characteristic as well as its susceptibility to cold, and we finally estimated the role of MCU. We confirmed previous knowledge that NCLX activity is cold-dependent [26], and we showed that it was mainly involved in the steady-state Ca^2+^ concentration outside mitochondria. Interestingly, NCLX activity plays a major role in steady-state [Ca^2+^] and is inversely correlated to CRC values in HM. Its role is milder in BM and the correlation with CRC values is not as strict as in HM (Supplemental Figure S5D). Mild cold showed a strong inhibiting activity on the Ca^2+^ uptake rate even in the presence of the NCLX inhibitor, in BM and HM in the narrow window of 37 to 25 °C. According to the key role of the MCU complex in the mitochondrial Ca^2+^ uptake rate (Supplemental Figure S5C), we, thus, inferred that the inhibition of the MCU complex by cold could explain this decrease in the Ca^2+^ uptake rate within the range (37–25 °C). The discrepancy in the range of temperatures at which MCU and NCLX are inhibited, (37–25 °C) and (37–15 °C), respectively, would have strong consequences on the net flux of Ca^2+^ and could participate in the increase in CRC value.

Our study has several limitations. First, we worked on intact mitochondria, not exposed to pathological processes such as I/R, but this was the only way to isolate cold as the sole factor influencing mitochondrial functions. Second, we studied non-cellular-specific brain and heart mitochondria. Indeed, brain and heart cells contain different types of mitochondria according to the different cell types, but, as used in clinical setting, therapeutic hypothermia is applied to the whole brain or heart, which is consistent with our approach. Third, our model excluded the effect of cell antioxidants and the effect of cold on them, but our model of isolated mitochondria was the only possible way to study the cold effects on MRC complexes.

In conclusion, we demonstrated that, in mixed substrates condition, cold uncouples differentially ROS production from respiration and mitochondrial Ca^2+^ fluxes, which could likely protect mitochondria from oxidation burst and mPTP opening, respectively.

## Figures and Tables

**Table 1:** Q_10_ values calculated, for respiration, ROS production rate or Calcium uptake rate in mitochondria from brain and heart. Temperature range have been determined by segment between characteristic inflexion points in Arrhenius plots. For Ros production, the ranges of temperature were [15–25 °C] and [25–37 °C] for GluMP and [15–20 °C] and [20–37 °C] for Succ. The highest Q10 values are shown in bold.

Mitochondria		Brain		Heart	
Respiration	Rotenone	GluMP	Succ	GluMP	Succ
6 to 37 °C	−	2.76		2.83	
	+		3.25		2.71
6 to 25 °C	−	**4.14**		**9.21**	
	+		**4.86**		**3.18**
25 to 37 °C	−	1.45		1.84	
	+		1.72		2.38
**ROS production**	**Rotenone**	**GluMP**	**Succ**	**GluMP**	**Succ**
6 to 37 °C	−	2.31	**3.41**	2.59	**4.68**
	+	3.00	2.55	3.10	2.03
6 to 15 °C	−	1.52	**13.26**	**4.96**	**25.93**
	+	**3.29**	2.28	**4.61**	3.01
15 to 20/25 °C	−	3.86	**4.11**	1.82	**15.58**
	+	2.05	**4.02**	**5.15**	2.59
20/25 to 37 °C	−	2.05	1.57	2.13	1.33
	+	2.12	2.36	1.51	1.53
**Ca2+ uptake rate**		**−CGP**	**+CGP**	**−CGP**	**+CGP**
6 to 37 °C		1.57	1.60	1.65	1.74
15 to 25 °C		1.05	1.43	1.08	1.08
25 to 31 °C		**3.32**	2.41	**3.81**	**4.61**
25 to 37 °C		2.19	1.77	2.34	2.60

## Supplementary Materials

The following supporting information can be downloaded at: https://www.mdpi.com/article/10.3390/cells11060989/s1, Figure S1. Counting and characterization of mitochondria in brain and heart samples. Figure S2. Cold-dependent effect on H_2_O_2_ detection by AmplexRed kit. Figure S3. CRC controls: effect of pyruvate addition, and of substrate fueling. Figure S4. Cold-dependent respiration and ROS production normalized by the count of mitochondria. Figure S5. True CRC calculation, calibration and Q10 coefficients. Figure S6. Calibration of TMRM fluorescence assay to estimate change in mitochondrial membrane potential (Ψm).

## Abbreviations

ADP	Adenosine Diphosphate
BM	Brain Mitochondria
BSA	Bovine Serum Albumin
CA	Cardiac arrest
CAC	Citric acid cycle
CRC	Ca^2+^ retention capacity
FET	Forward electron transport
Glu MP	Glutamate Malate Pyruvate
HM	Heart Mitochondria
I/R	Ischemia-reperfusion
MCU	Mitochondrial Ca^2+^ Uniporter
mPTP	Mitochondrial permeability transition pore
MRC	Mitochondrial respiratory chain
NADH	Nicotinamide Adenine Dinucleotide reduced
NCLX	Na^+^/Ca^2+^ exchanger
RET	Reverse electron transport
ROS	Reactive oxygen species
TH	Therapeutic hypothermia
