# Supplementary material for: Cooling Uncouples Differentially ROS Production from Respiration and Ca2+ Homeostasis Dynamic in Brain and Heart Mitochondria
Source: Cells. 2022 Mar 14;11(6):989. doi: 10.3390/cells11060989 (PMC8947173; doi:10.3390/cells11060989)
Supplement: Supplementary file 1 [file cells-11-00989-s001.zip › cells-1571844-supplementary.pdf]

Supplemental Figure

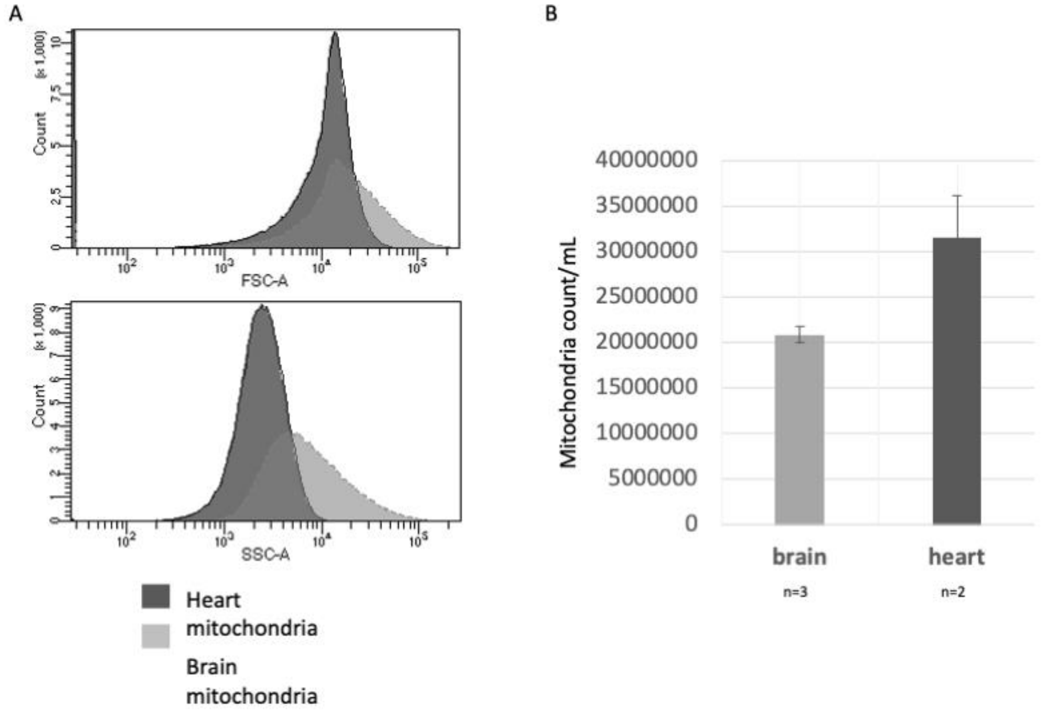

Figure S1. Counting and characterization of mitochondria in brain and heart samples. A, Histogram overlays which represent mitochondria morphology according to forward scatter (FSC-A) and side scatter (SSC-A) in brain and heart mitochondria samples. **B,** Mitochondria count in brain and heart samples (3 and 2 samples, respectively). Data are represented as mean ± 95%CI.

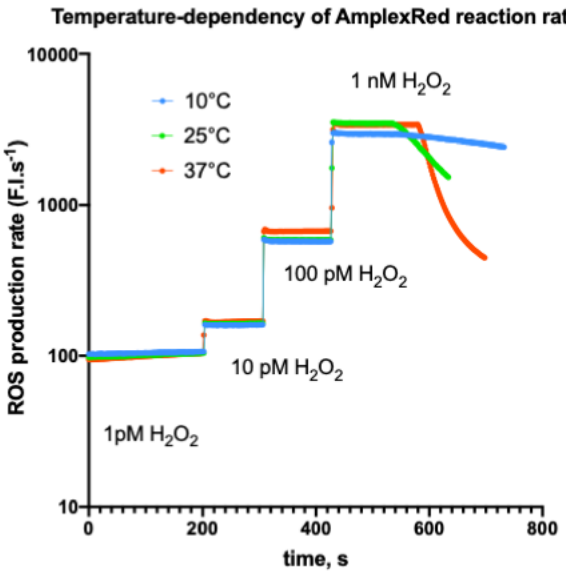

Figure S2. Cold-dependent effect on H_2O_2 detection by AmplexRed kit. ROS production rate in heart mitochondria, in arbitrary fluorescence unit/sec, at 10, 25 and 37°C. Increasing concentrations of H_2O_2 were added. N = 1.

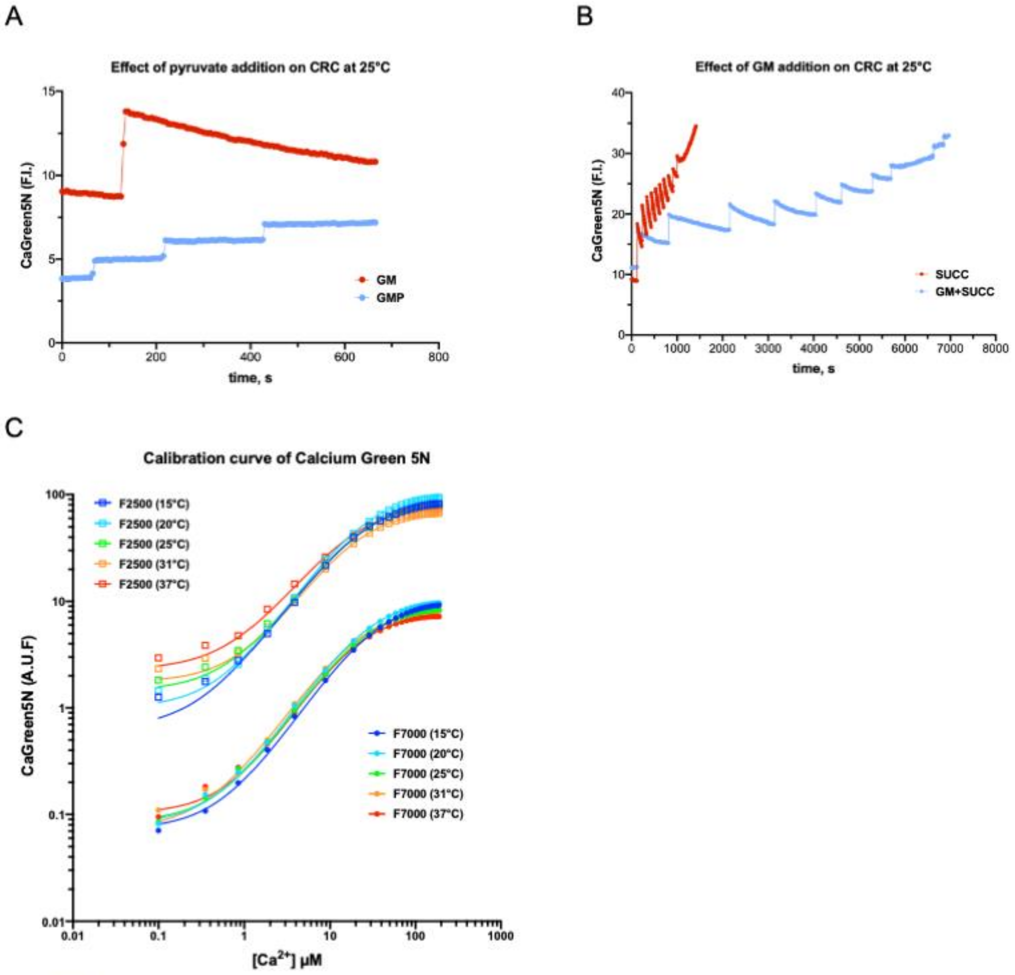

D

Nonlin fit<br>Table of results	A<br>F2500 (15°C)<br>Y	B<br>F7000 (15°C)<br>Y	C<br>F2500 (20°C)<br>Y	D<br>F7000 (20°C)<br>Y	E<br>F2500 (25°C)<br>Y	F<br>F7000 (25°C)<br>Y	G<br>F2500 (31°C)<br>Y	H<br>F7000 (31°C)<br>Y	I<br>F2500 (37°C)<br>Y	J<br>F7000 (37°C)<br>Y
**Sigmoidal, 4PL, X is cor**										
**Best-fit values**										
Bottom	0.6341	0.07274	0.9972	0.08124	1.443	0.08452	1.745	0.07158	2.277	0.1023
Top	90.25	10.37	102.3	10.62	89.90	9.071	72.68	8.902	81.94	7.760
IC50	24.36	32.92	24.41	26.42	24.41	24.51	20.92	21.99	18.65	20.82
HillSlope	1.142	1.223	1.213	1.234	1.174	1.229	1.209	1.191	1.135	1.271
logIC50	1.387	1.517	1.388	1.422	1.388	1.389	1.321	1.342	1.271	1.318
Span	89.61	10.29	101.3	10.54	88.46	8.987	70.94	8.830	79.66	7.658
**95% CI (profile likelihoo**										
Bottom	0.1089 to 1.153	0.03337 to 0.1118	0.4492 to 1.539	0.03342 to 0.1287	0.8548 to 2.022	0.04405 to 0.1247	1.110 to 2.370	0.01981 to 0.1228	1.203 to 3.319	0.04661 to 0.1573
Top	89.09 to 91.49	10.26 to 10.48	101.2 to 103.5	10.51 to 10.73	88.67 to 91.24	8.968 to 9.158	71.59 to 73.89	8.805 to 9.005	80.23 to 83.91	7.669 to 7.858
IC50	23.59 to 25.17	32.19 to 33.69	23.76 to 25.09	25.82 to 27.05	23.59 to 25.29	23.97 to 25.07	20.06 to 21.84	21.36 to 22.64	17.50 to 19.90	20.15 to 21.52
HillSlope	1.101 to 1.185	1.190 to 1.257	1.173 to 1.255	1.198 to 1.270	1.126 to 1.224	1.194 to 1.264	1.146 to 1.274	1.150 to 1.233	1.051 to 1.223	1.218 to 1.327
logIC50	1.373 to 1.401	1.508 to 1.528	1.376 to 1.399	1.412 to 1.432	1.373 to 1.403	1.380 to 1.399	1.302 to 1.339	1.330 to 1.355	1.243 to 1.299	1.304 to 1.333
**Goodness of Fit**										
Degrees of Freedom	21	21	21	21	21	21	21	21	21	21
R squared	0.9998	0.9999	0.9998	0.9999	0.9998	0.9999	0.9996	0.9998	0.9991	0.9997
Adjusted R squared	0.9998	0.9999	0.9998	0.9999	0.9997	0.9999	0.9995	0.9998	0.9990	0.9997
Sum of Squares	4.735	0.03029	5.418	0.04286	5.950	0.02999	6.846	0.04627	17.03	0.05596
Sy.x	0.4748	0.03798	0.5080	0.04518	0.5323	0.03779	0.5709	0.04694	0.9006	0.05162
RMSE	0.4442	0.03553	0.4751	0.04226	0.4979	0.03535	0.5341	0.04391	0.8424	0.04829

Figure S3. CRC controls: effect of pyruvate addition, and of substrate fueling. A, Representative CRC at 25°C showing the inhibitory effect of pyruvate addition on Ca^{2+} uptake by heart mitochondria. **B,** Representative CRC at 25°C showing the inhibitory effect of Glutamate + Malate (GM) addition on Ca^{2+} uptake by heart mitochondria. **C,** Calibration curves of Calcium Green 5N obtained on F2500 and F7000 spectrophotometers. Values

represent mean ± 95%CI. **D,** Results of the 4-component sigmoid fit with GraphPad Prism. Calculated Kd (EC50) Calcium Green 5N varied from 19 to 33μM depending on the temperature and the spectrofluorimeter.

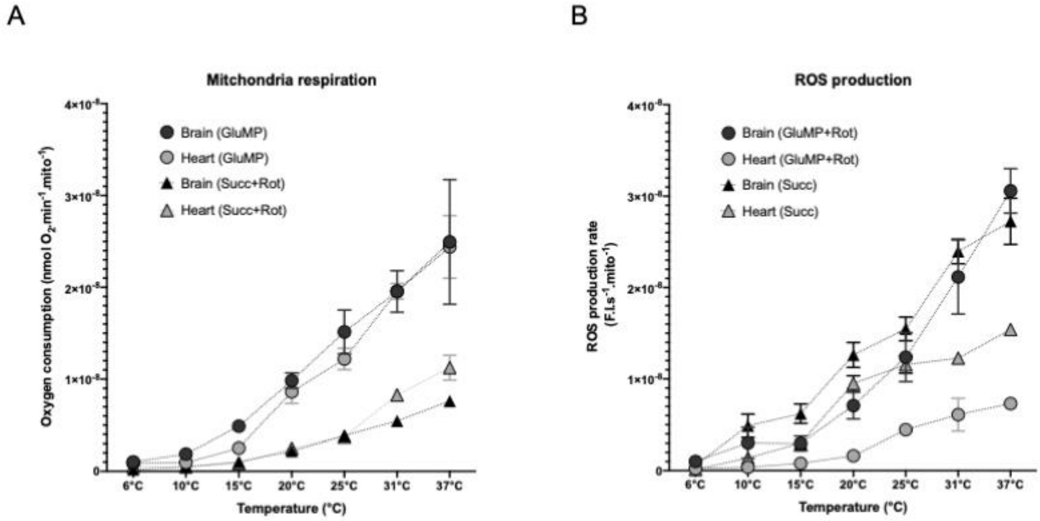

Figure S4. Cold-dependent respiration and ROS production normalized by the count of mitochondria. A, Mitochondrial oxygen consumption in brain and heart mitochondria, in nmol O_2/min/mitochondria, according to temperature (6, 10, 15, 20, 25, 31 and 37°C). Data are extracted from the figures 1A and 1B. **B,** ROS production rate in brain and heart mitochondria, in arbitrary fluorescence unit/sec/mitochondria, according to temperature (6, 10, 15, 20, 25, 31 and 37°C). Data are extracted from the figures 2A and 2B. Values represent mean ± 95%CI.

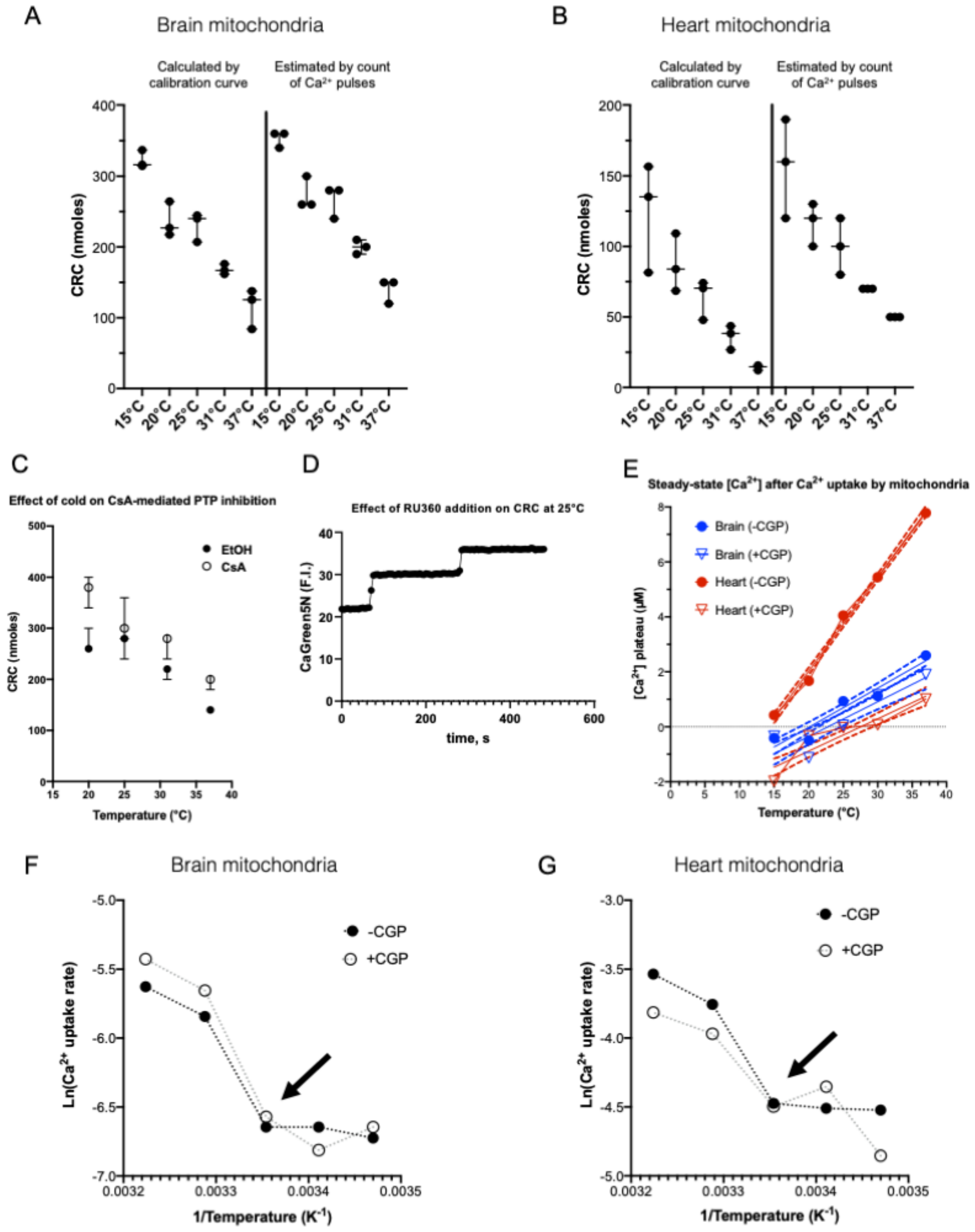

Figure S5. True CRC calculation, calibration and Q_{10} coefficients. A, Comparative dotplot representing the CRC values obtained by the Ca^{2+} pulse counting method (left) or by the calibration curve method (right) in brain mitochondria. Median ± 95%CI; n=3. **B,** as A in heart mitochondria; n=4. **C,** Ca^{2+} retention capacity (CRC) traces for brain in presence of Ethanol (EtOH) or Ethanol + Cyclosporine A (CsA). 2-way RM ANOVA showed a significant effect of temperature (p=0.0034), of CsA treatment (p=0.0247) without interaction between both factors

(p=0.2038). **D,** Representative CRC at 25°C showing the inhibitory effect of RU360, an inhibitor of MCU, on Ca^{2+} uptake by heart mitochondria. **E,** Effect of cold on the steady-state [Ca^{2+}] outside brain or heart mitochondria at equilibrium with or without addition of CGP-37157. Arrhenius plots of Ca2+ uptake rate with or without NCLX inhibitor, CGP-37157, in brain **(F)** and heart **(G)** mitochondria.

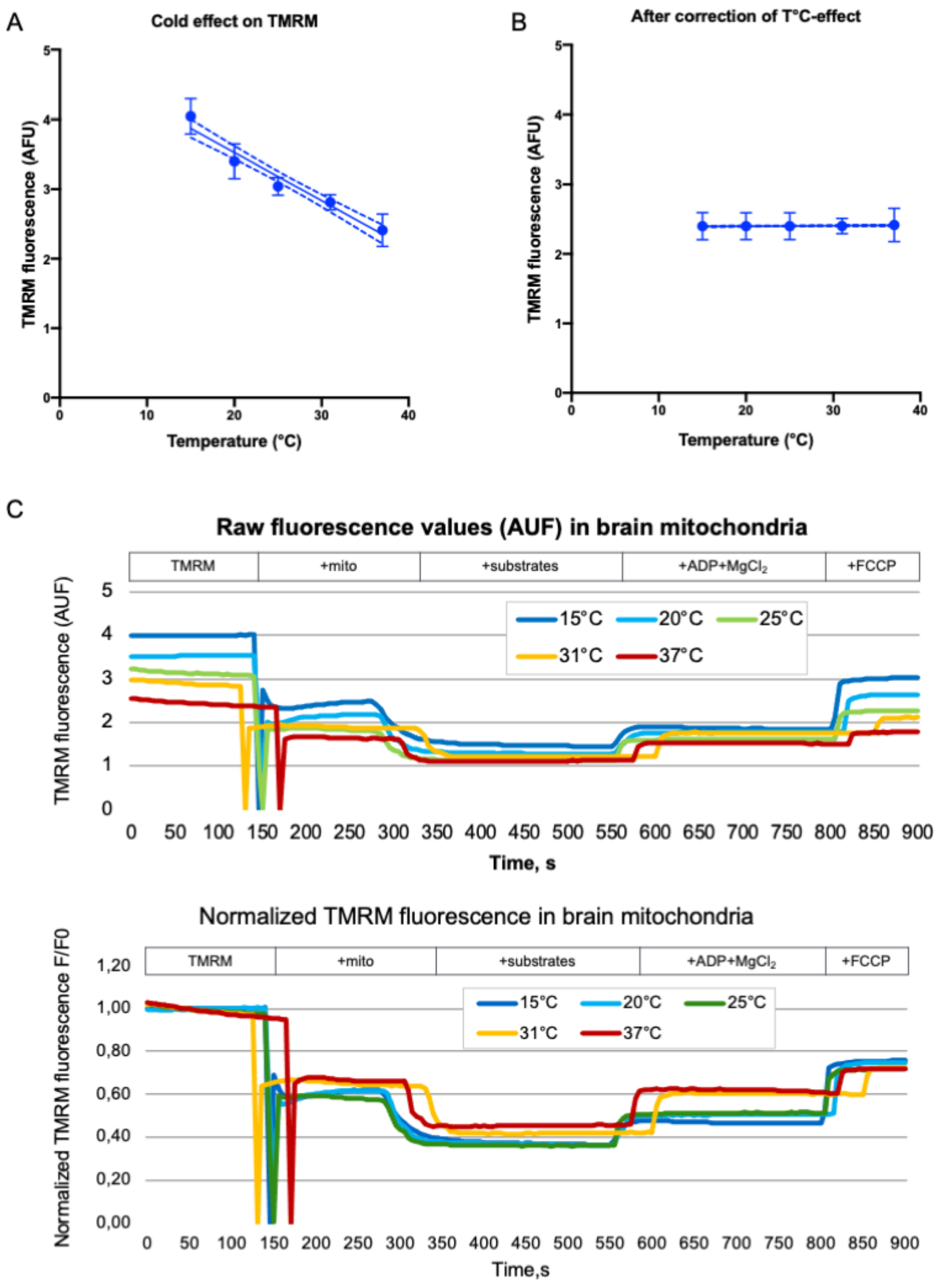

Figure S6. Calibration of TMRM fluorescence assay to estimate change in mitochondrial membrane potential (Ψm). A. Basal TMRM fluorescence in absence of mitochondria at 15, 20, 25, 31 and 37°C showed a strong cold-dependency. **B.** Correction factor applied to remove the cold-dependency on TMRM fluorescence. **C.** Example of a Ψm assay by TMRM fluorescence in brain mitochondria. After TMRM is added in the bath, mitochondria are added, then substrate (Glutamate, Malate and Succinate at its EC50 for maximal ROS production at each T°C) what led to the maximal hyperpolarisation step in the protocol (State 2 respiration). Thus, ADP + $MgCl_2$ was added to stimulate ATP synthase activity (Stat3 respiration) before 10µM FCCP was added to disrupt Ψm.
